# Bougie-Assisted Blakemore Tube Insertion: A Case Series

**DOI:** 10.5811/cpcem.65782

**Published:** 2026-10-30

**Authors:** Shane Gardner, Andrew Tachney, Adele Cobbs, Margaret Putman, Travis Hase

**Affiliations:** * Advocate Christ Medical Center, Department of Emergency Medicine, Oak Lawn, Illinois; † Northwestern University Feinberg School of Medicine, Department of Anesthesiology, Chicago, Illinois; ‡ The University of Chicago Pritzker School of Medicine, Department of Medicine, Section of Geriatrics and Palliative Medicine, Chicago, Illinois; § University of Illinois College of Medicine at Chicago, Department of Emergency Medicine, Chicago, Illinois; ¶ Wake Forest University School of Medicine, Department of Emergency Medicine, Winston-Salem, North Carolina

**Keywords:** Blakemore, bougie, gastrointestinal hemorrhage, varices, case series

## Abstract

**Introduction:**

Variceal hemorrhage is a life-threatening complication of portal hypertension that requires rapid hemorrhage control. Gastroesophageal balloon tamponade devices are effective temporizing measures when endoscopic or definitive therapies are delayed or unsuccessful, but placement can be technically challenging during active bleeding. A bougie-assisted technique has been proposed to improve ease and success of placement.

**Case Series:**

We describe three patients with cirrhosis and varices who presented with massive upper gastrointestinal bleeding and hemorrhagic shock. All received standard medical therapy and required escalation due to ongoing bleeding. In each case, a Sengstaken-Blakemore tube was placed using a bougie tracheal tube introducer as a semirigid stylet. The device was advanced under laryngoscopy through the esophagus and into the stomach without procedural complications. Hemostasis was achieved in all cases following balloon inflation, allowing hemodynamic stabilization and transition to definitive therapies. Two patients ultimately died due to complications of hypoxic-ischemic injury, while one survived to discharge. Use of the proximal gastric aspiration port appeared to reduce the risk of tip displacement compared to distal port insertion.

**Conclusion:**

Bougie-assisted gastroesophageal balloon tamponade device placement is a practical and effective modification for managing catastrophic upper gastrointestinal hemorrhage, and this approach may improve placement success and speed when standard technique has been unsuccessful. Wider adoption of this readily available adjunct could enhance procedural reliability, although further study is needed to better define outcomes and optimal technique.

## INTRODUCTION

Variceal hemorrhage is a life-threatening complication of portal hypertension, most commonly occurring in patients with advanced cirrhosis.^1^ Rapid control of bleeding is essential, as mortality remains high despite advances in medical and endoscopic therapies.^2^ When definitive intervention is delayed, unavailable, or unsuccessful, temporizing measures such as a gastroesophageal balloon tamponade may be lifesaving.^3^–^5^

Successful placement of gastroesophageal balloon tamponade devices, such as the Sengstaken–Blakemore tube, is technically challenging, particularly in the setting of ongoing hemorrhage and poor visualization. Specifically, the length and flexibility of the Blakemore contributes to a loss of directional control of its tip, commonly resulting in coiling and a failure to advance. Using a freezer or ice water bath may help to stiffen the tubing; however, this is time-consuming, and the effect is transient. Magill forceps may be used to grasp the tube and inch it forward, but this risks damage to the balloon and may injure surrounding tissues and does not mitigate the tendency for coiling.^4^ Additionally, several modified techniques using various guidewires have been described, but these approaches have not substantially improved ease or reliability of placement and may require unfamiliar or unavailable equipment.^6^–^8^ Bougie tracheal tube introducers are ubiquitous, and their use as a semirigid external stylet for placing a gastroesophageal balloon tamponade device has shown promise in simulation studies, improving both time to placement and first-pass success; however, in vivo experience remains limited.^9^, ^10^

We report three cases of massive upper gastrointestinal (GI) bleeding in which a bougie-assisted technique facilitated successful placement of a gastroesophageal balloon tamponade device. These cases highlight the safety and effectiveness of this modified approach, which offers a practical and readily available adjunct for managing catastrophic hemorrhage when conventional placement methods prove difficult.

## CASE SERIES

We describe three patients with varices who presented to the emergency department (ED) with massive upper GI bleeding and hemorrhagic shock requiring temporizing bleeding control. In all cases, a bougie tracheal tube introducer was used as a semirigid stylet to facilitate placement of a Blakemore tube.

### Case 1

A 62-year-old man with history of alcohol-related cirrhosis, hepatocellular carcinoma, portal vein thrombosis, and esophageal varices presented for altered mental status and hematemesis. Upon arrival, his heart rate was 46 beats per minute (bpm) and blood pressure (BP) was 82/64 mm Hg. On examination, he was unresponsive to noxious stimuli and had large-volume hematemesis with aspiration. The patient was intubated for airway protection, and standard therapies for suspected variceal bleeding were initiated, including ceftriaxone, pantoprazole, and octreotide. Persistent shock prompted initiation of a massive transfusion protocol, and vasopressors were started.

Due to uncontrolled hemorrhage and delay to endoscopy, a Blakemore tube was placed using a bougie-assisted technique. The bougie was inserted into the distal gastric aspiration port and used as a semirigid stylet to guide placement under video laryngoscopy. Once positioned, the gastric balloon was partially inflated, and the position was confirmed radiographically. The gastric balloon was then fully inflated, and the bougie withdrawn (Image 1).

*CPC-EM Capsule*

What do we already know about this clinical entity?

*Blakemore tubes are used to temporize uncontrolled upper gastrointestinal bleeding as a bridge to definitive care, but they are challenging to place.*

What makes this presentation of disease reportable?

*A simulation study of bougie-assisted Blakemore placement showed improved first-pass success and decreased placement time, but in vivo experience is limited.*

What is the major learning point?

*A bougie can serve as a semirigid external stylet to aid Blakemore placement. The added rigidity gives an advancing vector of force to the tip and prevents coiling.*

How might this improve emergency medicine practice?

*Bougie-assisted Blakemore placement is an effective rescue technique using a familiar and ubiquitous tool without an apparent increase in risk.*

The distal tip of the Blakemore was inadvertently withdrawn into the esophagus, likely related to use of the distal port. The gastric balloon remained appropriately positioned within the stomach. Minimal ongoing bleeding was noted from a preexisting gastric tube and a subsequently placed gastric tube terminating in the esophagus. The patient sufficiently stabilized to undergo endoscopic variceal band ligation and transjugular intrahepatic portosystemic shunt (TIPS) placement. His hospital course was complicated by the development of multiorgan failure and hypoxic-ischemic brain injury. The patient died on hospital day 11.

### Case 2

A 63-year-old man with alcohol-related cirrhosis complicated by ascites and esophageal varices presented with large-volume hematemesis. Upon arrival, his heart rate was 82 bpm, and BP was 77/53 mm Hg. Examination demonstrated ongoing hematemesis, scleral icterus, and abdominal ascites.

He was intubated, standard therapies for suspected variceal bleeding were initiated, and he was resuscitated with a massive transfusion protocol and vasopressors. While in the ED, emergent esophagogastroduodenoscopy (EGD) identified an actively bleeding distal esophageal varix that could not be successfully banded, and an esophageal stent was placed by the gastroenterology team. Unfortunately, bleeding recurred, prompting placement of a Blakemore tube for temporizing hemorrhage control. A bougie-assisted technique was again used under video laryngoscopy. In this case, the bougie was inserted into the proximal gastric aspiration port. Placement was straightforward and without complication (Image 2).

Despite inflation of the gastric balloon, bleeding from the esophagus persisted, prompting inflation of the esophageal balloon to a pressure of 34 mm Hg, which achieved hemostasis. After improvement in hemodynamics, the patient underwent successful embolization. Unfortunately, he developed severe hypoxic-ischemic neurologic injury and cerebral edema, and care was transitioned to comfort-focused measures. The patient died on hospital day 2.

### Case 3

A 48-year-old man with alcohol-related cirrhosis and varices was transferred with ongoing hemorrhage after endoscopic control of only one of two bleeding varices. Upon arrival, his heart rate was 110 bpm, and BP was 92/53 mm Hg. He had been intubated by the transferring facility, and standard therapies for variceal hemorrhage were ongoing, including resuscitation with blood products. In response to uncontrolled bleeding despite endoscopic therapies, a Blakemore tube was placed. A bougie-assisted technique was again employed as described in Case 2, using the proximal gastric aspiration port. Under direct laryngoscopy, the device was appropriately positioned without difficulty and successfully achieved hemostasis with inflation of the gastric balloon alone (Image 3).

The patient underwent a TIPS procedure, which was unsuccessful. He subsequently had coil embolization of two gastroesophageal varices. Repeat EGD showed three large varices, which were banded. After treatment of aspiration pneumonia and hepatic encephalopathy, he was discharged on hospital day 9.

## DISCUSSION

These cases highlight a practical, resource-conscious modification to a high-risk and technically challenging procedure used in the emergent management of life-threatening upper GI bleeding. Advancement of a Blakemore tube during active hemorrhage is frequently complicated by coiling, malposition, and placement delay related to multiple attempts, with risks including ongoing bleeding, procedural failure, airway compromise, and esophageal perforation.

Use of a bougie as a semirigid external stylet improves directional control and mitigates the tendency of the tube to coil or kink in the oropharynx and esophagus. Once inflated, the gastric balloon provides sufficient resistance to allow the bougie tip to dislodge from the side port, releasing the bougie while leaving the gastric tip appropriately positioned within the stomach. This observation aligns with emerging data, including simulation-based research demonstrating improved first-pass success and reduced placement time compared to standard technique under conditions mimicking massive upper GI hemorrhage and airway contamination.^9^

To place a Blakemore tube using this modified technique (Video):^4^

1. Prepare the gastroesophageal balloon tamponade device and check balloon inflation to ensure proper function. Fully deflate the balloons before insertion.
2. Assemble the unit by wedging the coudé tip of the bougie into the most proximal gastric aspiration side port of the Blakemore tube (Image 4).
3. Holding some tension, wrap the Blakemore a few times around the bougie and then lubricate the unit. This tension and added rigidity allow the operator to transmit an advancing vector of force to the tip of the Blakemore.
4. Use either direct or video laryngoscopy to lift the glottic structures anteriorly, ensure stability of the endotracheal tube, and allow exposure of the proximal esophagus.
5. Place the Blakemore-bougie unit into the esophagus and gently advance to a depth of approximately 50 cm or until there is resistance.
6. Inflate the gastric balloon with 50 mL of air and confirm appropriate positioning with a radiograph.
7. Inflate the gastric balloon fully with an additional 200 mL of air (total 250 mL).
8. Retract the unit gently until there is resistance, indicating the gastric balloon is abutting the gastroesophageal junction.
9. Apply gentle traction to only the bougie, dislodging the tip from the gastric aspiration port. There will be some slight resistance as the tip detaches from the side port.
10. Withdraw the bougie fully from the patient.
11. Place the device to 1 kg of traction.
12. Confirm final Blakemore and gastric balloon positioning with a radiograph.
13. Assess for and manage ongoing bleeding, inflating the esophageal balloon only if clinically indicated.

In all three cases, the bougie-assisted approach allowed rapid, controlled placement without procedural complications, and hemostasis was achieved in each instance. These findings are consistent with a prior series describing this modification, although that report was limited to a single Blakemore tube type.^10^ Notably, technique variation may be important. Use of the distal gastric aspiration port, as in Case 1, was associated with unintended withdrawal of the tube tip into the esophagus upon removal of the bougie. This is suboptimal because the gastric drainage ports are intended to decompress the stomach and monitor for ongoing gastric bleeding. In our case, a prepositioned orogastric tube allowed continued decompression and monitoring of gastric output, and the Blakemore’s gastric balloon positioning was unaffected; therefore, the displaced tip of the Blakemore was left in place. In contrast, use of the proximal gastric aspiration port in Cases 2 and 3 allowed reliable placement without displacement of the device tip during detachment of the bougie. This distinction may represent a simple technical optimization that improves procedural reliability. We hypothesize that using the proximal gastric aspiration port leaves a length of unattached, pliable tubing that cannot readily be drawn past the inflated gastric balloon into the esophagus.

Our experience underscores the importance of procedural adaptability when conventional methods prove inadequate. The ability to quickly modify a lifesaving intervention with readily available equipment, such as a bougie, may substantially influence patient outcomes when definitive therapy is delayed. Importantly, procedural success did not necessarily translate into survival, reflecting the severity of underlying disease and the high mortality associated with this condition. However, in each case, the gastroesophageal balloon tamponade achieved its intended role as a bridge to definitive therapy.

Although no complications occurred in these three cases, the risk profile of this modification is not fully characterized. Theoretical risks include injury to the glottis, esophagus, or stomach. Disruption of varices or displacement of previously placed hemorrhage-control measures is a risk with any instrumentation of the esophagus. Several factors mitigate these concerns, however. The bougie is specifically designed for insertion into the airway and is pliable. Theoretically, it should pose little added risk to esophageal tissue, particularly as the tip is shrouded within the soft, flexible tip of the Blakemore tube. In our experience, minimal force is required to advance the unit, and the improved directional control avoids forceful or repeat blind placement attempts as well as the introduction of more rigid tools such as Magill forceps. Balanced against the immediate threat of exsanguination, we believe the incremental risk of this technique is low and the risk-benefit balance favorable.

Limitations include a small sample size, retrospective observation, and inability to compare outcomes directly with standard placement technique. The bougie-assisted approach was used as the initial placement method in all three cases rather than as a rescue after failed conventional placement, so no comparative conclusions regarding standard technique can be drawn. Assessment of placement ease is operator-reported and neither standardized nor objective and may reflect operator experience rather than the technique itself.

## CONCLUSION

This case series adds to a limited but growing body of evidence supporting bougie-assisted gastroesophageal balloon tamponade device placement as an effective, accessible, and potentially more reliable modification to traditional technique. It expands prior published experience by demonstrating reproducible use across multiple cases and identifies a potential technical advantage of the proximal gastric aspiration port. Although further study is needed to define success rates, complications, and patient-centered outcomes compared with conventional technique, the infrequent use of gastroesophageal balloon tamponade devices will likely impose methodologic challenges to future investigation. By bridging the gap between simulation-based findings and clinical application, this series emphasizes the value of readily available adjuncts in the management of catastrophic upper GI hemorrhage. Although promising, there are insufficient data and experience to suggest a bougie-assisted technique be first-line when gastroesophageal balloon tamponade placement is indicated. While an attempt using standard technique is advisable, familiarity with bougie-assisted Blakemore placement as a rescue tool may prove beneficial.

## Supplementary Information

Video Instructional video demonstrating bougie-assisted Blakemore tube placement on a task training model.

## Figures and Tables

**Image 1:**
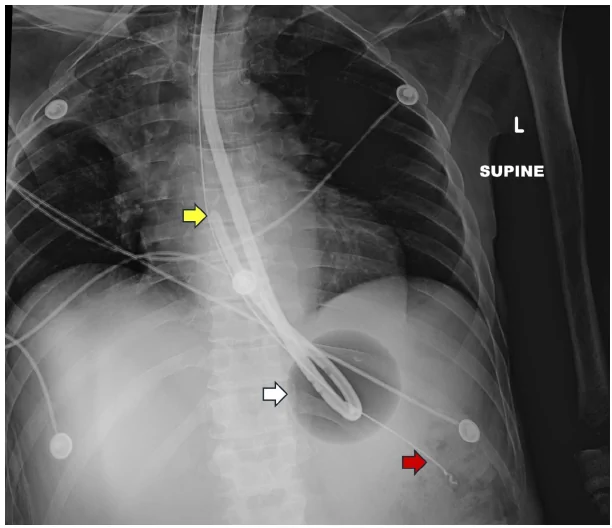
Sengstaken-Blakemore tube placement. The gastric balloon (white arrow) is inflated within the stomach, but the tip (yellow arrow) has been inadvertently withdrawn into the esophagus. The companion gastric tube (red arrow) remains correctly positioned within the stomach.

**Image 2:**
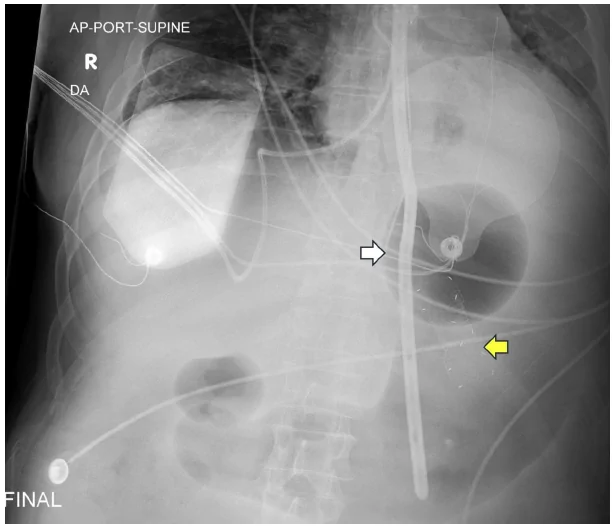
Sengstaken-Blakemore tube placement. The gastric balloon (white arrow) is inflated within the stomach. A previously placed esophageal stent is displaced into the stomach (yellow arrow).

**Image 3:**
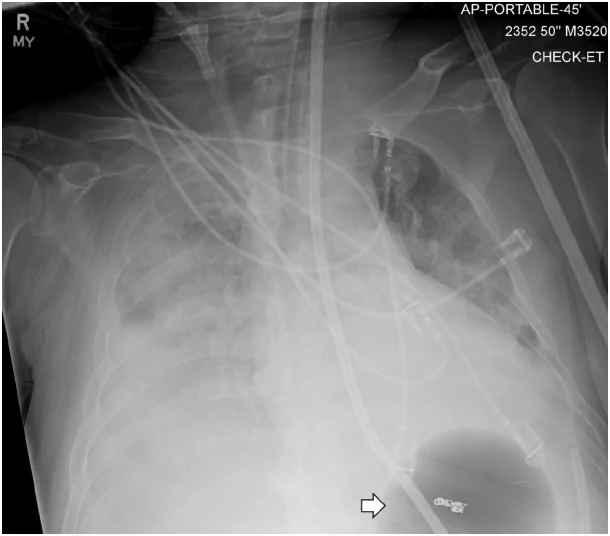
Sengstaken-Blakemore tube placement. White arrow indicates the gastric balloon inflated within the stomach.

**Image 4:**
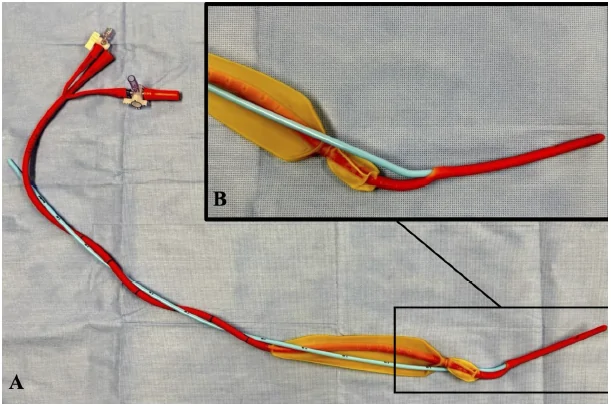
Unit assembly for bougie-assisted Sengstaken-Blakemore tube placement. A, With gastric and esophageal balloons fully deflated, the coudé tip of the bougie (tracheal tube introducer) is wedged into the most proximal gastric aspiration port. The tube is wrapped around the bougie to provide rigidity and directional control. B, Magnified view showing the attachment point where the bougie tip is wedged approximately 0.5 centimeters into the gastric aspiration port.
